# Influence of Ethyl Caproate on the Size of Lipid Vesicles and Yeast Cells

**DOI:** 10.3390/biomimetics5020016

**Published:** 2020-04-27

**Authors:** Tsuyoshi Yoda, Akira Ogura, Tomoaki Saito

**Affiliations:** Aomori Prefectural Industrial Technology Research Center, Hirosaki Industrial Research Institute, 1-1-8 Ougi-Machi, Hirosaki City, Aomori 036-8104, Japan; akira_ogura@aomori-itc.or.jp (A.O.); tomoaki_saito@aomori-itc.or.jp (T.S.)

**Keywords:** microscopic observation, lipid vesicles, ethyl caproate, size distribution, yeast, *Saccharomyces cerevisiae*

## Abstract

Ethyl caproate (EC) is a key flavor component of sake. Recently, in sake brewing, an effort has been underway to increase the content of aromatic components such as EC. However, the function of EC in yeast cells remains poorly understood. Therefore, we investigated the effects of EC using cell-sized lipid vesicles. We found that vesicle size decreases in a concentration-dependent manner when EC is contained in lipid vesicles. Furthermore, yeast experiments showed that a strain producing high quantities of EC in its stationary phase decreased in size during EC production. Given caproic acid’s (CA) status as the esterification precursor of EC in yeast, we also compared lipid vesicles containing CA with those containing EC. We found that CA vesicles were smaller than EC vesicles of the same concentration. These results suggest that EC production may function apparently to maintain cell size.

## 1. Introduction

Ethyl caproate (EC) is known as a key flavor component of not only sake but also various other fermented beverages [1,2,3,4]. Although EC’s flavor function is well known, EC has not been researched with respect to other functions, particularly its physiological function. For example, some molecules affect cell membrane properties based on their molecular interactions with membranes [5]. Recently, cell-sized lipid vesicles (>10 μm) have been actively studied as cell membrane models [6]. These vesicles have membrane structures similar to those of cells with regard to size and membrane composition [7]. Since they are large enough, we can observe them directly via microscopic observation, as has been done in studies of many other organic compounds, such as capsaicin [6], cholesterol derivatives [8,9], and anesthetics [10,11]. Previous studies have reported that aroma compounds such as EC may influence yeast cell membranes, but a cell membrane function for EC has not been researched [12]. At present, there is limited information on membranes containing EC, whose production in yeast results from the condensation of caproic acid (CA) and ethanol. Differences between EC and CA are important for determining their membrane functions due to differences in their chemical structures and hydrophobicity. Yeast with high EC productivity tends to also produce a large amount of CA. In order to reveal the role and mechanism of EC with respect to membranes, we also undertook a comparative investigation of the properties of membranes containing CA. CA has a bad smell. Therefore, CA is not only a precursor of EC but also has negative effects on the quality of beverages.

In this work, we used an unsaturated lipid 1,2-dioleoyl-sn-glycero-3-phosphocholine (DOPC). DOPC is one of the most general lipids for model membrane experimental system, and thus, we have reported changes in membrane properties using DOPC lipid vesicles plus other biofunctional molecules [6,7,8,9,10,11]. DOPC has been used as a model lipid for yeast membranes previously [13,14]. Vanegas reported that inclusion of the unsaturated lipid provides a model system that, in comparison to previous studies, more closely resembles a real yeast plasma membrane [14]. Although we did not find any reports that testify that yeast has DOPC, an article reported that yeast has both oleic acids and phosphatidylcholine, which are components of DOPC [15]. During previous studies, we have researched the interaction of biological molecules, such as capsaicin [6], oxidized cholesterol [8,9,16], local anesthetics [10,11] proteins [16], polyphenols [17], between membranes lipids. In these studies, there were some approaches, mainly using lipid vesicles. In the method, lipids were rehydrated together with objective molecules or compounds to make lipids vesicles and observed. We obtained valuable results using these methods, as reported before. Therefore, in the present study, we used the same methods.

Our institute has developed the yeast strain Saccharomyces cerevisiae, Mahoroba-Gin, which shows the ability to produce high levels of EC [18,19]. The sake made using the strain has ~10–12 ppm EC. A sake production company has reported that sake made using normal strains of yeast has 1.4 ppm EC [20]. Therefore, our developed strain has enough EC for a comparative investigation with a normal yeast strain.

Here, we investigated the presence and effects of EC on membranes, not only revealing EC’s flavor. We undertook this investigation by constructing and observing cell-sized lipid membrane vesicles containing EC.

## 2. Materials and Methods

DOPC, CA, chloroform, yeast nitrogen-based (YNB) medium without glucose or ammonium sulfate were obtained from Tokyo Chemical Industry Co Ltd. (Japan), Aldrich (USA), Kanto-Chemical (Japan), and Difco Laboratories (USA). The EC, glucose, and ammonium sulfate were purchased from Wako Pure Chemical (Japan). Ultrapure water obtained from a Millipore Milli-Q purification system (Millipore, Bedford, MA, USA) was used for reagent preparation and cleaning of glassware.

### 2.1. Preparation of Lipid Vesicles

For the lipid vesicles (giant unilamellar vesicles (GUVs); model membranes/liposomes), we used a slightly modified version of the method of natural swelling from dry lipid films outlined in our previous study [21]. Lipid mixtures (DOPC only or DOPC with EC or CA) were dissolved in chloroform in a glass test tube under nitrogen gas. They were dried under vacuum for 3 h to form thin lipid films. The films were then hydrated overnight with ultrapure water. The final concentration of the hydrated film was 0.2 mM lipid.

### 2.2. Confirmation of Presence of EC in Lipid Vesicles by Gas Chromatography–Mass Spectrometry

**Figure 1:**
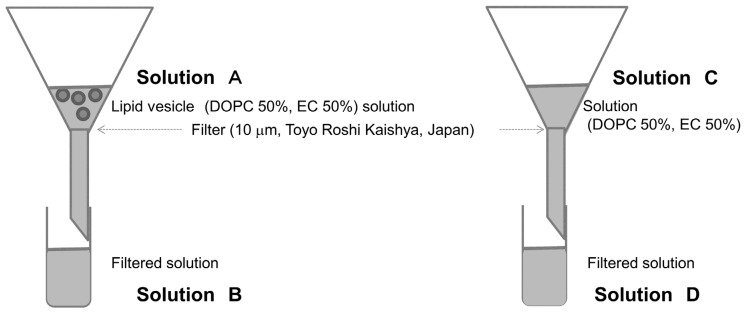
Experiment to confirm presence of EC in membranes using a filter.

Gas chromatography–mass spectrometry (GC/MS) was performed using a 7890B GC System/5977A MSD (Agilent Technologies, Inc., United States) and 10 μm filters (Toyo Roshi Kaisha Ltd., Japan) to determine the presence of EC in lipid vesicles. Methyl caproate was added as internal standard to the 50% EC lipid vesicle solution (solution A in Figure 1), and sodium chloride was measured using stir-bar sorptive extraction [22]. To prevent EC presence out of the membranes, the filter was caught because it is not possible to simply attach a filter even if the EC is present in the solution before it reaches the membranes. As a comparison, we made a solution by mixing EC and DOPC (solution C in Figure 1). When preparing these solutions, sodium chloride was added to promote extraction [22]. Each experiment was conducted three times.

### 2.3. Yeast Culturing

In the comparative analysis of normal and high-EC yeast strains, Mahoroba-Hana was used as the normal yeast strain, and Mahoroba-Gin was used as the high-EC yeast strain [18,19]. These yeasts were prepared under two conditions, slant and stationary phases, because the stationary phase contains yeast in a relatively homogeneous phase and may have relatively high EC content. To induce the stationary phase in each yeast strain, a static culture was used, with all yeast extracted in the same lot. The timing of observation at the stationary phase was determined a priori based on the growth character of each yeast strain: two days post extraction for Mahoroba-Hana and three days post extraction for Mahoroba-Gin. The trends of growth curve of each strain grown in synthetic YNB medium (prepared using nitrogen-based medium without glucose or ammonium sulfate (Difco, United States), glucose (Nacalai Tesque, Japan), and ammonium sulfate (Wako, Japan),) were also confirmed. Growth curves were obtained from OD 660 measurement using UV spectrometer UV-1800 (Shimadzu, Japan).

### 2.4. Microscopic Observation

**Figure 2:**
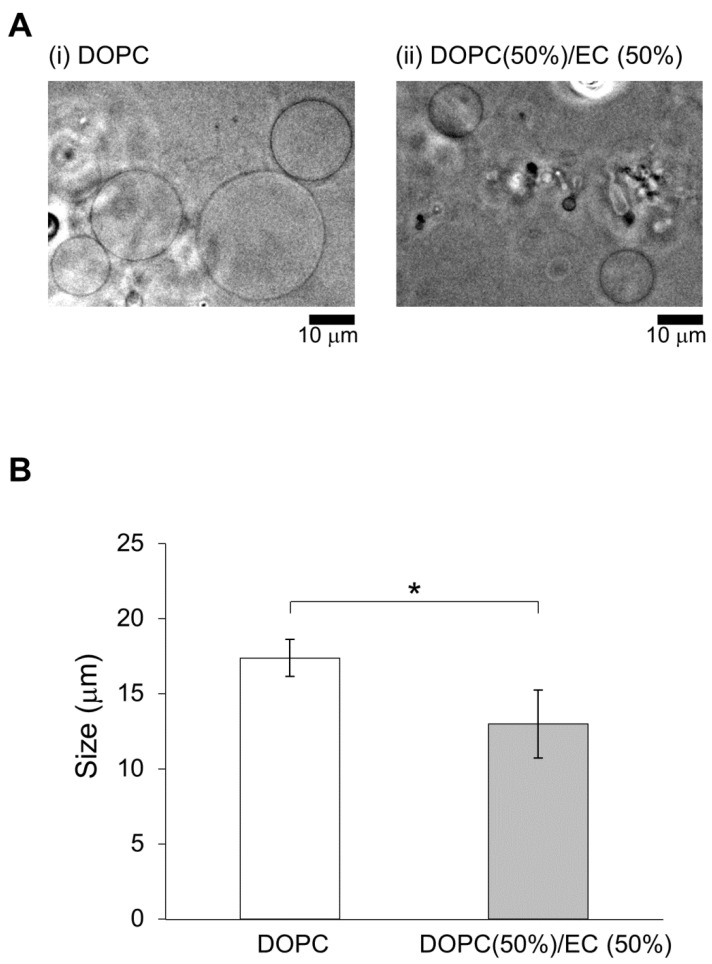
(**A**) Images of a typical lipid vesicles captured using a phase-contrast microscope: (i) DOPC, (ii) DOPC (50%) with EC (50%). The scale bars are 10 μm. (**B**) Vesicle size based on contents; white represents DOPC vesicles, and gray represents vesicles filled with 50% DOPC and 50% EC. (* *p* < 0.05).

**Figure 3:**
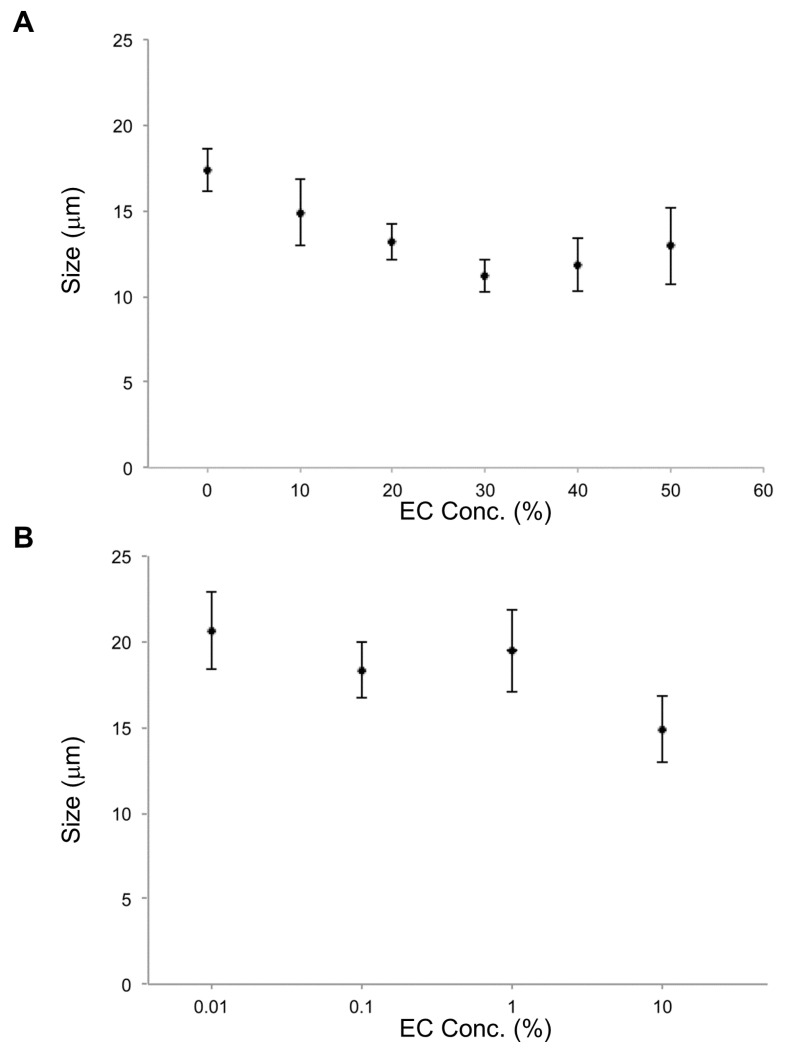
Size of lipid vesicles made from membranes containing ethyl caproate (EC) at different concentrations: (**A**) 0% to 50%, (**B**) 0.01% to 10%.

**Figure 4:**
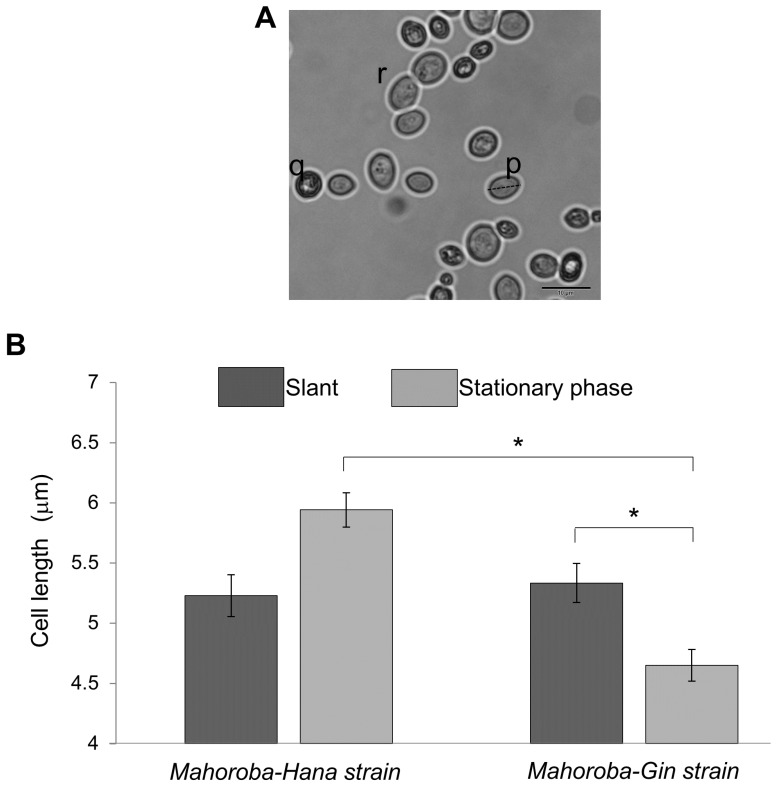
Sizes of yeast strain cells in two situations. (**A**) Typical images of yeast. Only alive cells (such as those labeled (p)) are counted. Cells in budding (such as those labeled (r)) were also excluded from the count. (**B**) Size distributions. Dark gray represents yeast in slant state, and light gray represents yeast in stationary phase. Over 30 cells were counted for the calculation of average size, with standard errors shown as error bars. (* *p* < 0.05).

Size trends in lipid vesicles and yeasts were then observed using a microscope (BX53, Olympus, Japan). DOPC, DOPC/EC, and DOPC/CA membrane systems, together with the size distribution for each lipid vesicle type, were investigated. At least 30 lipid vesicles were observed for each type. Sphere lipid vesicles were chosen randomly, and sizes were measured as diameters (Figure 2 and Figure 3). The yeast strains were compared to define the maximum yeast cell length using the following software: cellSens Standard 2 (Olympus, Japan) and ImageJ (downloaded from https://imagej.nih.gov/ij/download.html). Yeast cell size trends were based on counts and measurements of cells stained with methylene blue and observed under a microscope (Olympus BX53, Japan). Dead cells (such as (q) in Figure 4A) were not counted. Budding cells (such as (r) in Figure 4A) were also excluded because size and count were too difficult to objectively define for budding cells. It was confirmed that the relative ratio of budding vs non-budding cells was not significantly different between Mahoroba-Hana and Mahoroba-Gin strains. These exclusions still left at least 30 yeast cells for counting and measurement. Average and standard errors of size were calculated and then summarized.

## 3. Results

### 3.1. Effect of Ethyl Caproate on Size of Lipid Vesicles

Here, we investigated the presence and effects of EC on membranes to not only reveal EC’s flavor mechanism but also elucidate EC’s physiological function. We conducted this investigation by constructing and observing cell-sized lipid membrane vesicles containing EC. EC presence in lipid vesicles was confirmed by GC/MS.

First, two types of lipid vesicles were prepared: DOPC vesicles and 50% DOPC/50% EC vesicles. In a previous study, membrane dynamics of 50% DOPC/50% cholesterol vesicles were investigated under oxidative stress to realize the effect of containing of cholesterol [21] because 50% cholesterol is almost the maximum content to form a cell-sized vesicle. Similarly, 50% was considered better EC content to reveal effect on physiological function.

Lipid vesicles were smaller for membranes containing EC/DOPC than for membranes containing DOPC only (Figure 2). The concentration was determined to be 3.33 ± 0.35mM using GC/MS with a standard solution (solution A at Figure 1). The concentration was smaller than expected based on a priori calculations (10.0 mM, the value was calculated using solution concentration). The disparity may have been caused by evaporation during the construction of lipid vesicles or during GC/MS preparation and measurement. The concentration of filtered solution A (solution B) was 1.32 ± 0.06 mM. The EC concentration detected for the prepared EC/DOPC solution (solution C) was the same as was used for the preparation of lipid vesicles: 5.34 ± 0.14 mM. EC was also detected in solution D, a 4.23 ± 0.10 mM concentrate of filtered solution C (Figure 1). About 60% of EC contents were deleted from the lipid vesicle solution using filters. On the other hand, only about 20% of EC contents were deleted from the solution. These results indicate that many of the lipid vesicles containing EC were over 10 μm in diameter and were therefore intercepted by the filter (Figure 1). For this reason, lipid vesicle concentrations were reduced in the filtered solution from their concentrations in the pre-filtered solution, though few EC-containing vesicles were in solution to begin with.

In the investigation of effects of EC concentration on vesicle size, the smallest average size occurred at 30% EC. In cases of EC concentration at 10%, 20%, 30%, 40%, and 50%, lipid vesicles with EC were smaller than lipid vesicles without EC. For EC concentrations under 10%, EC content effects on size were not observed. According to these results, EC concentrations over 10% affect the size of DOPC lipid vesicles (Figure 3).

### 3.2. Effect of Cell Size on Productivity of Strains of Yeast-Dependent EC

**Figure 5:**
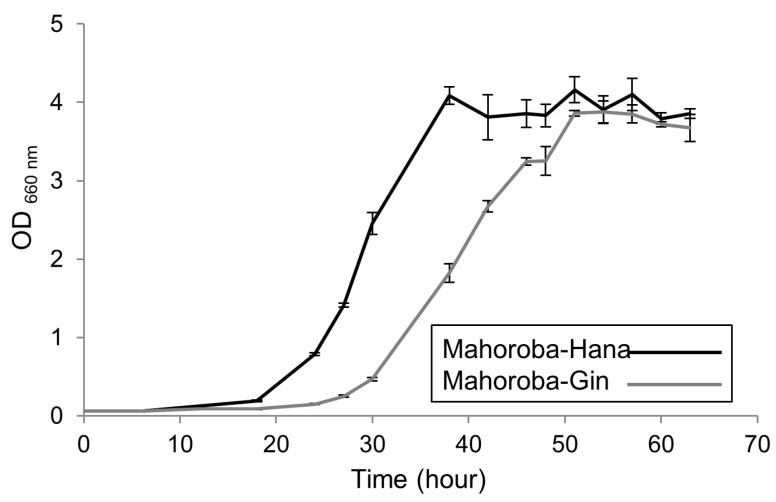
Growth curve of two yeast strains. Black shows Mohoroba-Hana strain and grey shows Mohoroba-Gin strain, each with OD = 660 nm.

The trends of growth curve of each strain grown in synthetic YNB medium are shown in Figure 5. At the slant state, sizes of these two types of yeast were almost equal (Figure 4B, dark gray bar). In the case of Mahoroba-Hana, the size at stationary phase was increased. In contrast, Mahoroba-Gin size at stationary phase was reduced (Figure 4B, light gray). Our institute reported previously that Mahoroba-Gin has higher productivity than Mahoroba-Hana, with values of EC concentration as 5.0 and 1.3 ppm, respectively, at the same condition [18,19]. The size trend in Mahoroba-Gin, which has high EC productivity capability, was similar to that of lipid vesicles as they increased in EC concentration from 10% to 30%. The stationary phase may increase EC concentration vs. the slant state in Mahoroba-Gin. The results suggest that increasing EC content affects membrane properties, leading to reducing vesicle size.

### 3.3. Effect of Caproic Acid on Size of Lipid Vesicles

**Figure 6:**
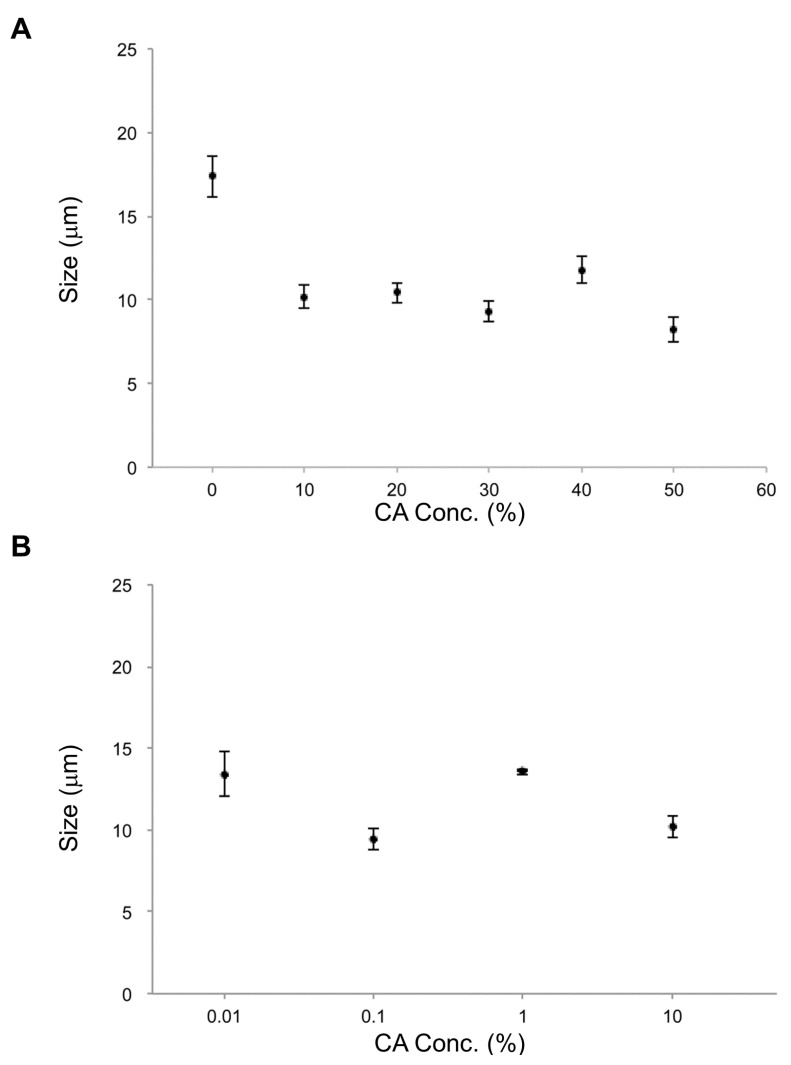
Size of lipid vesicles made from membranes containing caproic acid (CA) at various concentrations: (**A**) 0% to 50%, (**B**) 0.01% to 10%. Standard errors are shown as error bars.

In the parallel investigation of CA effects, it was found that the smallest average vesicle size occurred at 50% CA. For all CA concentrations measured (0.01% to 50%), lipid vesicles containing CA were smaller than those without CA (Figure 6), just as for EC. Furthermore, lipid vesicles containing CA at each concentration were smaller than those containing EC at that same concentration. CA apparently has a strong effect on size reduction for lipid vesicles. CA’s strong membrane effect may be related to not only its smaller molecular size compared with that of EC but also different amphiphilicity that will affect vesicle formation, particularly at higher CA/EC concentrations. The previous study compared the effect of size by decreasing two types of flavonoids, epi-gallocatechin gallate (EGCG) and theaflavin [17]. The experiment differs from the present study; the previous study only added the flavonoids solution after making the lipid vesicle from DOPC, and the size decreased depending on the time. The mechanisms to form small sizes of lipid vesicles might almost be the same. We concluded that the phenomena caused by inserting flavonoids depended on their molecular size and hydrophobicity for lipid packing in the previous study [17]. This suggests that the mechanisms of small lipid vesicles forming in the presence of EC or CA might be caused by an interplay between their sizes and shapes, as well as the molecular packing in the membranes. CA has been reported in the transport membranes of cell-sized lipid vesicles containing Poly(dimethylsiloxane) [23]. It is also reported that CA has functions in tomato plants against *Botrytis cinerea* [24]. Therefore, CA and membrane interaction may have significance. The report also indicates that CA influences signal transduction in tomato. Signal transduction may influence membranes’ physical and chemical properties, such as fluidity and permeability [6]. According to these studies and our experimental results, CA and membrane interaction may have important biological roles.

Production of EC by esterification of CA may be considered to have an effect of preventing destabilization of yeast cell size. CA is known to be a byproduct of production of longer acyl chain in lipid production.

### 3.4. Role of EC in Membranes

Because EC is important for sake quality, the EC productivity of yeast strains has been researched using several methods. One popular method of estimation of EC productivity involves the analysis of fatty acid synthase (FAS) [1,25]. In the yeast cell, long chain fatty acids are produced for purposes such as membrane construction. EC is formed by esterification using caproic acid as a precursor. CA is mainly biosynthesized using acetyl-CoA and malonyl-CoA as substrates in the fatty acid synthesis pathway of sake yeast [1]. Therefore, it is considered that CA and EC are produced as by-products in the process of synthesizing long-chain fatty acids in yeast. FAS mutations then lead to the production of numerous short- and medium-length acyl chain molecules such as CA, rather than long acyl chains. EC is then produced from CA and ethanol, but actual EC productivity is typically confirmed by small-scale brewing. Both FAS analysis and small-scale brewing require expensive equipment and substantial time.

On the other hand, our microscopic observation detection method may be a candidate tool for direct estimation of EC production. Our method is based on lipid metabolic pathways and rigorous size determination of yeast cells. Only the longest distance of cell p was measured as cell length and compared in the present study, even though this does not provide enough information for a detailed evaluation. Therefore, this method should be developed as a tool for evaluation not only of yeast cell size but also of yeast cell structural parameters [26]. Using this tool, these authors revealed the target genes of the compounds of interest as well as other genes involved with functionally related cellular pathways [27].

Molecular interaction mechanisms for the results described above are drawn from previous studies [17,28,29] and proceed as follows: hydrophobic strength and tight packing increase curvature, reducing the radius of the lipid molecules and of the vesicles. The shape of lipid vesicles is determined by the correlation among optimal surface area *a0*, hydrocarbon volume *v*, and critical chain length *lc*, called the shape factor *v/a0lc*. Smaller phospholipid shape factors, or more conical molecules, resulted in the formation of more curved and smaller lipid vesicles. Therefore, the EC and CA could be inserted in the lipid bilayers while the lipid vesicles are forming. As a result, the complex of two phospholipids with these molecules and a central was more conical for CA than EC. Size reduction in lipid vesicles and yeast may indeed occur via such mechanisms, because our results exhibit identical trends. Our work suggests the following: EC exists in lipid vesicle solution according to GC/MS. EC causes strong packing of lipid molecules and reduces the radii of vesicles and cells. Research will continue on the investigation of properties of membrane containing EC to observe shape transformations under stresses such as osmotic [30], heat [8,9] and so far, to reveal molecular interaction of EC with lipids molecules. Notably, we have to confirm the EC presence in yeast cells, whether it is in the membranes or at other places in the yeast cells.

Finally, the present study has investigated just two strains of yeast differentiated by EC productivity. The comparative study we undertook remains to be generalized more systematically across strains and conditions. Much additional research will be needed in order to continue to improve high-quality sake with flavor contributions from contents such as EC. The structural factors promoting EC productivity in yeast strains remain to be fully revealed. Furthermore, the size-based detection EC productivity capability will be of great benefit for screening yeast strains for good EC productivity for sake.

In this first report of cell-sized lipid vesicles containing DOPC with EC, we found that increasing EC content affects membrane properties, leading to reduced vesicle size. EC does appear to be located at the membrane and does affect the size of lipid vesicles. On the basis of comparative analysis of cell size and EC in yeast strains, EC production appears to similarly affect the size of actual cells via interactions with membranes. Comparative measurements of vesicle size for vesicles containing CA vs. EC suggest that the production of EC by esterification of CA may have the effect of preventing destabilization of yeast cell size.
